# Single-nucleus RNA sequencing reveals the specific molecular signatures of myeloid cells responding to brain injury after microglial replacement

**DOI:** 10.3389/fimmu.2025.1625673

**Published:** 2025-07-24

**Authors:** Xiao-Yi Xiong, Haicheng Yuan, Ying Mu, Yi He, Fang Xie, Xiao-Shuang Feng, Jia-Xin Xie, Xin-Ru Pan, Yu-Fei Wang, Jian Gong, Xiaoming Zheng, Peng-Fei Wang

**Affiliations:** ^1^ Acupuncture and Tuina School, Chengdu University of Traditional Chinese Medicine, Chengdu, China; ^2^ Key Laboratory of Acupuncture for Senile Disease (Chengdu University of Traditional Chinese Medicine (TCM)), Ministry of Education, Chengdu, China; ^3^ Acupuncture and Chronobiology Key Laboratory of Sichuan Province, Chengdu, China; ^4^ The Second Department of Neurology, Qingdao Central Hospital, University of Health and Rehabilitation Sciences (Qingdao Central Hospital), Qingdao, China; ^5^ Department of Clinical Laboratory, Qingdao Central Hospital, University of Health and Rehabilitation Sciences (Qingdao Central Hospital), Qingdao, China; ^6^ Department of Neurology, Linyi People’s Hospital, Linyi, China; ^7^ Department of Anesthesia Surgery, Linyi People’s Hospital, Linyi, China; ^8^ Department of Neurology, Weihai Municipal Hospital, Cheeloo College of Medicine, Shandong University, Weihai, China

**Keywords:** brain injury, microglial replacement, myeloid cells, snRNA-seq, intracerebral hemorrhage

## Abstract

**Background:**

Myeloid cells, such as resident microglia (MG), infiltrating monocytes (Mo), macrophages (MΦ), and CNS border-associated macrophages (BAM) in the brain, participate in aged brain injury. Aged microglial replacement is protective against brain injury in aged mice; however, whether/how the molecular changes in myeloid cells are affected by this replacement in injured brains remains unclear.

**Methods:**

Aged microglia in mice were eliminated by PLX3397 for 21 consecutive days and repopulated following withdrawal for 21 days; then, intracerebral hemorrhage (ICH) models were constructed. Then, a single-nucleus transcriptomic analysis of acutely injured brains in aged mice with microglial replacement was performed.

**Results:**

We observed similarities but strong divergence in the composition and molecular change features of myeloid cells between the replacement (Rep) and control (Con) groups, indicating retention of the core transcriptome and development of differential genes in myeloid cells after microglial replacement in response to brain injury. Both MG and Mo/MΦ experience modification of immune responses after microglial repopulation, with more prominent changes in MG. Gene Ontology (GO) analysis showed that one term directly related to the “immune response” was shared between upregulated genes in Rep-MG and Rep-Mo/MΦ, while the other terms related to immune functions and other biofunctions were different between Rep-MG and Rep-Mo/MΦ, indicative of significantly different immune responses to brain injury between MG and MΦ. Furthermore, the trajectory analysis showed a significant transition from aged to young state in Rep-MG compared to only a modest youthful shift in Rep-Mo/MΦ, suggesting a rejuvenation process of aged microglia and macrophages toward young ones in response to brain injury after the treatment of microglial replacement.

**Conclusion:**

Our data indicate that microglial replacement-induced changes in the molecular heterogeneity and state transition of myeloid cells may be neuroprotective against acute brain injury.

## 1 Introduction

Intracerebral hemorrhage (ICH), a devastating subtype of stroke with high rates of mortality and morbidity, causes patients suffering devastating and debilitating neurological impairments that usually cannot fully recover due to no effective therapy (1). The prevalence of ICH could rise with the continues growing elderly population since advanced age has been shown as a major risk factor of ICH, such as contributing to the risk of developing ICH and poor functional outcomes (1), which may be related to the fact that the molecular level changes in the aging brain after ICH are largely unknown.

One of the critical aggravators of neural injury following ICH is neuroinflammation. Once ICH occurs, resident microglia quickly respond by transitioning into reactive states, and together with infiltrating periphery immune cells leads to a complex immune response in the brain (2–5). However, these processes become more quick in the aged brains, then an exaggerated and uncontrolled inflammatory phenotype becomes a hall marker of aged brain insults (6), which is largely related to the aged microglia with a primed profile by augmenting the production of inflammatory factors and chemokines following injury (7), resulting in more infiltration of peripheral immune cells. Microglia during aging experience pronounced phenotypic and functional changes, including altered gene expression (8–10) and exaggerated response to inflammatory stimuli (11), reminding us that resetting them to non-reactive homeostatic ones may be beneficial to functional recovery. For example, withdrawal of CSF1R inhibitors in the aged brain induces complete repopulation of young-like microglia , which exhibit characteristics resembling their youthful counterparts and reduce neuronal injury in response to brain insults (12, 13). However, it remains poorly understood whether and to what extent the molecular changes in young-resembling microglia occur in response to aged brain injury, and how these changes influence other myeloid cells.

Recently, single-cell and single-nucleus RNA sequencing (sc/snRNA-seq) technologies have enhanced the understanding of the molecular changes in brain disorders (e.g., stroke, neurodegenerative diseases) at single cell level. Although mounting single-cell transcriptomic studies have revealed remarkable immune diversity in the brain during aging and in certain brain disorders (14–20), little is known about the transcriptional changes in the myeloid cells after aged microglial replacement in response to ICH.

In this study, we aim to decipher and gain deeper insights into the impact of aged microglial replacement on the transcriptional diversity of the myeloid cells in aged brains in response to brain injury by performing the single-nucleus RNA-sequencing (snRNA-seq) analysis. This study demonstrates considerable cellular and molecular heterogeneity of myeloid cells [microglia (MG), monocytes/macrophages (Mo/MΦ), and border-associated macrophages (BAMs)] in injured brains caused by intracerebral hemorrhage (ICH) after microglial replacement and will provide some novel potential therapeutic targets derived from young-resembling microglia in aged mice , which may inform strategies to modulate microglial states in human aging-related health and disease.

## 2 Materials and methods

### 2.1 Animals

Male C57BL/6 mice (16–18 months) were purchased from Gempharmatech (Chengdu, China). All mice were housed under pathogen-free conditions with a 12-hour light/dark cycle, ambient temperature maintained at 25°C, and relative humidity of 45-55%, with ad libitum access to food and water. All animal experiments were conducted in accordance with the National Institutes of Health Guide for the Care and Use of Laboratory Animals in China approved by the Committee on the Ethics of Animal Experiments of Chengdu University of Traditional Chinese Medicine. The study complies with the ARRIVE (Animal Research: Reporting *in vivo* Experiments) guidelines. No abuse or maltreatment occurred during our study.

### 2.2 Compound and drug administration

PLX3397 (Catalog #S7817, Selleckchem Inc, Houston, TX) was resolved in a solution containing 5% DMSO (Corning, Catalog #02616006) and 95% Phosphate Buffered Saline (PBS; Biotopped Life Sciences, Cat# TOP1010-2L). Groups of mice received PLX3397 (80 mg/kg/day) for 21 consecutive days to eliminate microglia as previously described (13, 21) and then followed by withdrawal for 21 days before ICH induction. Mice in control groups received the same solution containing 5% DMSO and 95% PBS.

### 2.3 Collagenase-induced mouse model of ICH and neurological assessment

ICH was induced by injection of bacterial collagenase in mice as previously described (22, 23). Briefly, mice were fixed on a stereotaxic apparatus (RWD Life Science Co., Shenzhen, China) after being anesthetized with 3% isoflurane for anesthesia induction, and with 1.5% isoflurane for maintenance. A volume of 0.5 μL of 0.075 IU type VII collagenase (Sigma Aldrich) was injected into the left striatum (0.8 mm anterior and 2 mm lateral to bregma, at a depth of 3.5 mm) at a rate of 6.25 × 10^-^² μL/min using a microinfusion pump (Hamilton, Bonaduz, AG). The needle was removed after a 10 min pause following infusion and the burr hole was filled with bone wax. During and after the surgical operation, the body temperature of the mice was maintained at 37 ± 0.5°C by an electrical blanket. Before obtaining the brain tissues for snRNA-seq, the Zea-longa scores of ICH mice were assessed at days 1 after ICH by at least 2 investigators blinded to treatment.

### 2.4 Tissue dissociation

One day after ICH, the perihematomal brain tissues (24) from aged ICH mice were enzymatically dissociated to obtain single-nucleus suspensions using either a self-prepared hydrolysate or a commercial dissociation kit (MACS Mouse Tumor Dissociation Kit, DS_130-096-730). The enzymatic reaction was terminated by adding Hank’s Balanced Salt Solution (HBSS) containing calcium and magnesium (Gibco, Germany). The resultant cell suspension was sequentially filtered through 70-μm and 40-μm cell strainers, followed by centrifugation at 300 × g and 4°C for 10 minutes. Myelin debris was removed via Percoll gradient centrifugation (22%). Briefly, cells were suspended in 25 mL Percoll solution [18.9 mL gradient buffer (containing 5.65 mM NaH2PO4·H2O, 20 mM Na2HPO4·2H2O, 135 mM NaCl, 5 mM KCl, 10 mM glucose, pH 7.4); 5.5 mL Percoll (GE Healthcare, Germany); 0.6 mL 1.5 M NaCl], overlaid with 5 mL PBS, and centrifuged at 950 × g for 20 min at 4°C, without acceleration and brakes.

### 2.5 Single-nucleus RNA sequencing

Cell quantity and viability were assessed immediately post-sorting. A volume equivalent to 10,000 target cells was processed for library preparation. Gel bead-in-emulsions (GEMs) and sequencing libraries were generated using the Chromium Controller (10x Genomics) with Single-Cell Gene Expression v2/v3 Chemistry (for experiments comparing Aged + Control + ICH *vs*. Aged + Replacement + ICH; see **Supplementary Figure 2**; DG1000 Genomics, BIOMARKER Technologies, China), following the manufacturer’s protocol (Chromium Single-Cell 3′ Reagent Kits v2/v3 User Guide). Library quality and quantity were evaluated using a High-Sensitivity DNA Kit (Agilent Technologies, USA) on a 2100 Bioanalyzer (Agilent Technologies, USA). Sequencing was performed on a BGI DNBSEQ-T7 platform with rapid-run flow cells and paired-end 150-bp (PE150) reads.

### 2.6 Single-nucleus RNA-seq data preprocessing and normalization

Raw sequencing data (BCL files) were demultiplexed and converted to FASTQ format using BSCMatrix (DG1000 Genomics). Reads were aligned to the mouse reference genome GRCm38 (mm10; Ensembl) and quantified with BSCMatrix (http://www.bmkmanu.com/portfolio/tools). CellRanger identified 80,692 cells, with median values of 1,329 detected genes and 2,993 UMIs per cell. Data were analyzed in R using Seurat v4 (25, 26), with default parameters unless specified. Low-quality cells were filtered by excluding those with <100 UMIs, <500 or >7,000 genes, or >20% mitochondrial transcripts. Post-filtering, 68,489 cells remained. Gene expression was normalized using total transcript counts per cell scaled by a default factor and log-transformed (LogNormalize method). The 2,000 most variable genes were selected via variance-stabilizing transformation (vst). To enhance cell-type identification, these genes were supplemented with known markers for neural/immune cells and cell cycle regulators (27), which did not alter conclusions.

### 2.7 Identification of myeloid cells

A Seurat v4 approach (25) was used to integrate the data from corresponding samples, with three biological replicates for each group (Aged + Control + ICH and Aged + Replacement + ICH). Firstly, 3000 integration anchors (i.e., cells that are mutual nearest neighbors between replicates) were found to avoid results that fit too closely to a particular data set and therefore possibly failing to fit additional data. Then, these anchors were used as an input for the data integration procedure. As described in a corresponding vignette [https://satijalab.org/seurat/v3.0/cell_cycle_vignette.html], the integrated data were scaled, and unwanted sources of variation-namely the total number of counts per cell, the percentage of transcripts of mitochondrial genes per cell, and cell cycle effect-were regressed out. A principal component analysis was used for data dimensionality reduction, and the first 20 principal components were used in downstream analyses. Then, an unsupervised, graph-based approach with the resolution parameter set to 0.3 was performed to cluster the expression profiles for each condition separately. Two-dimensional t-SNE (Visualizing data using t-SNE) was used to visualize the clustering results. Based on the expression of reported/canonical markers, the clusters dominated by myeloid cells were identified and further analyzed for two conditions.

### 2.8 Comparative analysis

The comparative analysis was based on the raw counts but limited to the above-selected profiles and genes. For such a merged data set, variance stabilizing transformation (“vst”) was used to identify a new set of the 3000 most highly variable genes, which was further expanded by adding genes involved in the regulation of the cell cycle. As described above, we performed the computation of expression estimations, regression of unwanted variation, and data dimensionality reduction. Next, using the same approach as above, the expression profiles were clustered but with a resolution parameter set to 0.6. Then, two-dimensional UMAP was used to visualize the data after clustering. Based on the expression of reported/canonical markers of myeloid cells, clusters with cells of interest (microglia, macrophages, and BAMs) were identified. Differentially upregulated genes (signature genes) were found for each of the identity classes of interest. Next, a Wilcoxon rank-sum test implemented in Seurat v4 (min.pct = 0.25, only.pos = TRUE) was used to identify significantly upregulated genes between the compared groups. These genes were subsequently used for functional analysis and the characterization of the identified clusters. GO analysis was performed using the clusterProfiler v4.4.4 package (28).

### 2.9 Trajectory inference and analysis using Monocle3

Monocle3 (29, 30) was applied to further infer the changing process for the microglia or macrophage clusters induced by microglial replacement (n = 45070 nuclei) generated in Seurat. Monocle3 uses a dimensionality reduction approach to place individual cells in a two-dimensional space, removes batch effects through mutual nearest neighbor alignment, and connects single cells to build a trajectory in a semisupervised manner. Then, we used the integrated Seurat object with no further batch correction or dimensionality reduction in Monocle3 for clustering microglia or macrophages. We subsetted the microglia and macrophage clusters and programmatically specified the root of the trajectory by selecting the node most enriched for young cells. The trajectory and its direction calculated by Monocle3 agreed with the distribution of cells across the two conditions. Spatial differential expression analysis along the trajectory was performed using Moran’s I test in Monocle3, and genes with q < 0.05 were selected as trajectory-dependent (17083 and 13377 genes for microglia and macrophages, respectively). The set of genes was grouped into seven modules according to their RVAE decoded expression (31) along the trajectory.

### 2.10 Functional enrichment analysis

Functional enrichment analysis of trajectory-dependent genes (t-DEGs; microglia: n = 17,083 genes; macrophages: n = 13,377 genes) was performed using the clusterProfiler v4.4.4 package (28). Gene Ontology (GO) terms were annotated against the following databases: GO_Biological_Process_2018 , GO_Cellular_Component_2018 , and GO_Molecular_Function_2018 . Significant terms were filtered at a threshold of Padj < 0.05 (Benjamini–Hochberg correction) and compiled into module-specific lists (labeled 7_modules_q_moranI; see **Supplementary Tables 1**, **2** for microglia and macrophages, respectively). The top 10 most significant terms (*P* < 0.05) per module were visualized as dot plots. To decode temporal expression patterns of t-DEGs along pseudotime trajectories, we implemented the RVAE (Recurrent Variational Autoencoder) algorithm via the RVAgene Python package (v1.0; Python v3.9.6, PyTorch v1.9.0). Gene expression values were averaged within pseudotime bins, rescaled to the range [−1, 1], and used as input. The neural network architecture consisted of two symmetrical hidden layers (48 nodes/layer) and a two-dimensional latent space. Reconstructed trajectories from the output layer were visualized as heatmaps.

## 3 Results

### 3.1 Single-nucleus RNA-sequencing identifies neural and immune cells with distinct expression profiles upon brain injury after aging microglial replacement

To replace microglia in the aged brain, we fed 16-18-month old mice [termed aged mice (13)] with a CSF1R inhibitor PLX3397 by gavage for three weeks, followed by withdrawal of PLX3397 for an additional three weeks (13) (**Supplementary Figure S1A**). Then, the acute brain injury model of mice with ICH was established. Twenty-four hours after ICH, we found that the aged ICH mice with microglial replacement (Replacement) showed better neurofunctions when compared to those without microglial replacement (Control) (**Supplementary Figure S1B**), further confirming the neuroprotection of microglial replacement for aged brain injury of ICH (13).

**Figure 1:**
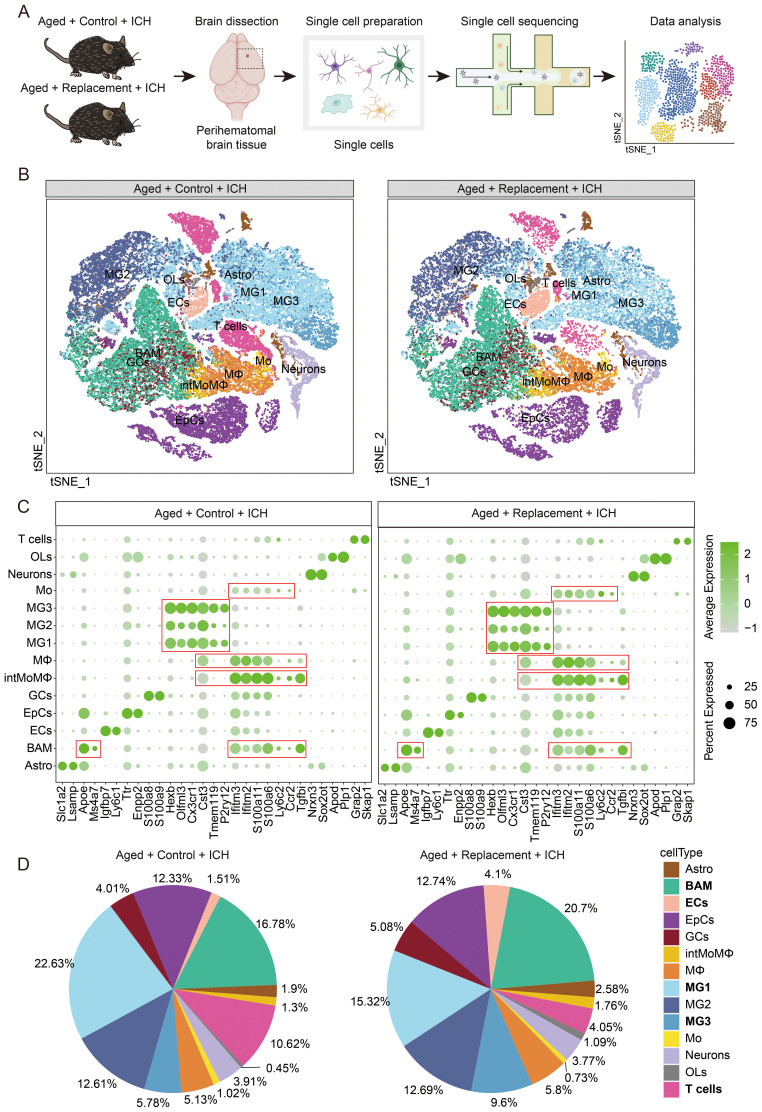
Identification of immune cell populations in control and tumor-bearing brain hemispheres. **(A)** Scheme of the experimental workflow. The used brain image was modified from the Database Center for Life Science. **(B)** t-SNE plot demonstrating clustering obtained from injured brains in aging ICH mice treated without or with microglial replacement groups (Aged + Control + ICH *vs* Aged + Replacement + ICH), three biological replicates were combined. Clusters annotations: Astro astrocytes, BAM CNS border-associated macrophages, ECs endothelial cells, EpCs epithelial cells, GCs granulocytes, MG microglia, Mo monocytes, intMoMΦ intermediate monocyte-macrophage, MΦ macrophages, OLs oligodendrocytes, and T cells T lymphocytes. **(C)** Expression of signature genes for identification of a cluster cell type. **(D)** Pie charts showing the distribution of the identified cell types across samples.

Then, the single-nucleus RNA sequencing (snRNA-seq) was performed to reveal the perihematomal brain tissues of aged ICH mice (**Figure 1A**; **Supplementary Figure S2**) and single-nucleus transcriptomic profiles for 40811 and 27678 cells in the injured brain without or with microglia replacement were selected for the analysis, respectively (**Supplementary Figure S3**). Given that myeloid cells participate in regulating brain injury (32–34). Then, we further visually inspected the transcriptomic diversity of computed clusters and dissected the clusters of myeloid cells by applying the immune cell marker panel (**Figure 1C**) created with the literature-based markers (35) and projecting the data onto two dimensions by t-distributed stochastic neighbor embedding (*t*-SNE) (**Figure 1B**).

To dissect the functional meaning of gene expression underlying microglial clusters (MG), we explored the available information on microglial phenotypes. All 3 clusters of MG were obtained in aged ICH mice with or without microglial replacement. The clusters of MG1–3 in both groups are characterized by relatively high expression of microglia-enriched genes (*Hexb*, *Cx3cr1*, *Tmem119*, and *P2ry12*) may partly reflect a signature of homeostatic microglia (HomMG). Furthermore, we identified three clusters of infiltrating monocytes/macrophages (Mo/MΦ) that could be further characterized by an inflammatory monocyte signature-Mo (Ly6c2^high^, Ccr2^high^, and Tgfbi^low^), an intermediate state of monocyte and macrophage signature-intMoMΦ (Ly6c2^high^ and Tgfbi^high^), and a differentiated macrophage signature-MΦ (Ly6c2^low^, Ifitm2^high^, Ifitm3-^high^, and S100a6^high^) (35) (**Figure 1C**). A high expression of Apoe in BAM, a kind of brain-resident self-renewing myeloid cells, is a major marker that can be distinguished from other myeloid cells (**Figure 1C**). These brain intrinsic and infiltrated peripheral myeloid cells identified in both two conditions represent similar but significantly different expression features and transient and intermediate activation states of myeloid cells.

The identified myeloid cells comprised the vast majority of all sorted cells (65.25% and 66.6% in brain injury without or with microglial replacement, respectively), whereas the proportion of identified myeloid cells varied between the two groups (**Figure 1D**). For example, the proportion of MG1 decreased from 22.63% in Control group to 15.32% in Replacement group. On the contrary, the proportion of MG3 increased from 5.78% to 9.6% in the injured brain after microglial replacement. In addition, the proportion of BAMs increased from 16.78% to 20.7% in injured brains after microglial repopulation (**Figure 1D**).

### 3.2 Assessment of molecular features that distinguish myeloid cells

**Figure 2:**
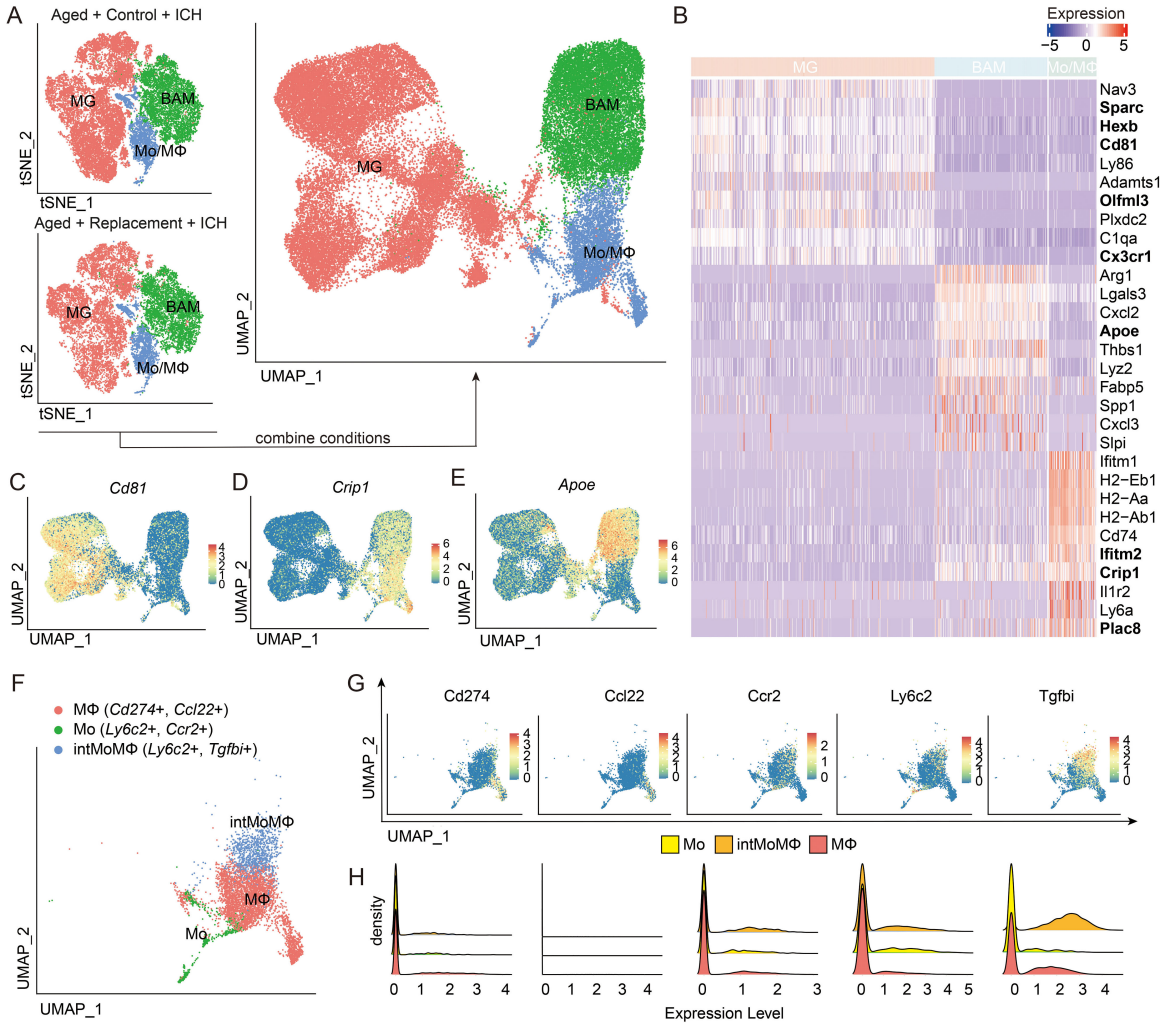
Transcriptomic characterization of main myeloid subpopulations. **(A)** Projection of cells combined from clusters identified as microglia (MG), monocytes/macrophages (Mo/MΦ), and BAMs from Aged + Control + ICH and Aged + Replacement + ICH groups. **(B)** The top ten differentially expressed genes (DEGs) for the three main identified cell populations, and new marker candidates are in bold. **(C–E)** Feature plots depicting genes highly expressed in MG **(C)**, Mo/MΦ **(D)**, and BAM **(E)**. **(F)** UMAP plot discriminating monocytes (Mo), monocyte-macrophage intermediate (intMoMΦ), and macrophage (MΦ) subpopulations. **(G, H)** Feature and density plots depicting the distribution and expression level of the marker genes, Cd274, Ccl22, Ccr2, Ly6c2, and Tgfb1, in **(F)**.

To identify the molecular features that distinguish myeloid cells in response to brain injury under the condition of aging microglial replacement, we extracted only the cells identified as MG, Mo/MΦ, and BAMs from all the conditions and replicates. The combined three cell subpopulations formed three separate groups after projecting on the two-dimensional space using a UMAP algorithm (**Figure 2A**). To confirm cell identities, we performed differential expression analyses between three subpopulations. Among the most highly upregulated genes in each group, we found the well-known microglial genes-*Sparc*, *Hexb*, *Cd81*, *Olfml3*, *Adamts1*, *C1qa*, and *Cx3cr1* (36–41) in MG, BAM genes-*Arg1*, *Lgals3*, *Cxcl2*, *Apoe*, *Thbs1*, *Lyz2*, *Fabp5*, *Spp1*, *Cxcl3*, and *Slpi* in BAMs, and Mo/MΦ genes-*Ifitm1*, *H2-Eb1*, *H2-Aa*, *H2-Ab1*, *Cd74*, *Ifitm2*, *Crip1*, *Il1r2*, *Ly6a*, and *Plac8* in Mo/MΦ (**Figure 2B**). Among these genes, such as *Fabp5*, *Spp1*, and *Arg1*, has been found in stroke-associated macrophages, foamy macrophages in atherosclerotic plaques, and lipid-associated macrophages in myocardial infarct (42–44). Spp1 and Lgals3 are related to the clearance of damaged cells and tissue repair (19, 45), Fabp5 and Lgals3 are related to lipid metabolism (46), *H2-Aa*, *H2-Ab1*, *H2-Eb1*, and *Cd74* are major histocompatibility complex (MHC) class II genes, Ifitm1 and Ifitm2 are ISG signature genes, and Plac8 is related to blood monocytes (47).

The expression of *Nav3*, *Sparc*, *Hexb*, *Cd81*, *Ly86*, *Adamts1*, *Olfml3*, *Plxdc2*, *C1qa*, and *Cx3cr1* was enriched only in MG (**Figure 2B**). Highly expressed genes in BAMs were *Arg1*, *Lgals3*, *Cxcl2*, *Apoe*, *Lyz2*, *Fabp5*, and *Spp1*. In addition, *Thbs1*, *Cxcl3*, and *Slpi* were highly expressed in only a small fraction of BAM. However, we found Lgals3 also appeared in a substantial fraction of Mo/MΦ and Fabp5 also found highly expressed by a fraction of Mo/MΦ, suggesting that these genes are not exclusive for BAMs in injured brains. For Mo/MΦ, we found enriched expression of previously reported genes and novel genes, such as Ifitm1, MHC-II genes (*H2-Eb1*, *H2-Aa*, and *H2-Ab1*), *Cd74*, *Il1r2*, *Plac8*, and *Ly6a* (48, 49) (**Figure 2B**). We then aimed to identify markers for the separation of MG and Mo/MΦ in injured brains. From the top differentially expressed genes (DEGs, ranked by the average log fold-change value, all with adjusted (Bonferroni correction) P-value < 10^−100^) in the MG and Mo/MΦ groups (**Figure 2B**), we selected candidate genes with enriched expression in a majority of cells in the group of interest-*Cd81* (MG), *Crip1* (Mo/MΦ) and *Apoe* (BAM; **Figures 2C–E**). Cd81, a member of the tetraspanin family of proteins, its up-regulation in reactive MG is thought to be involved in the glial response to CNS injury (38, 50). Cysteine-rich protein 1 (Crip1) expression in MΦ and Mo has been shown to participate in the regulation of the immune response (51, 52), and has an correlation with an increased risk for stroke (53). Apoe has recently been proposed as a marker of CNS BAM (40) and ApoE deficiency in mice caused a significant increase in infarct size and brain swelling (54).

Among the highly up-regulated genes in myeloid cells, we further divided these cells with previously reported markers into Mo (Ly6c2+, Ccr2+), intMoMΦ (Ly6c2+, Tgfbi+), and MΦ (Cd274+, Ccl22+) (35), which were also pronounced discrete in the UMAP results of brain injury with the largest proportion for macrophages, followed by monocyte/macrophage intermediate cells (intMoMΦ) and monocytes (**Figure 2F**). However, we found that except the expression of Tgfbi was almost in the intMoMΦ and Cd274 expressed in a small population of macrophages, the remaining reported markers were low expressed and limited mostly in corresponding myeloid cells (**Figures 2G, H**). In addition, we found a population of MΦ expressing Ccl5 genes, encoding chemokines important for T-cell recruitment (55, 56) (**Supplementary Figure S4**). Such expression pattern suggests different response signatures of myeloid cells in mediating the immune response of brain injury from aged ICH mice.

### 3.3 Distinct gene expression profiles of brain injury-associated microglia and monocytes/macrophages

**Figure 3:**
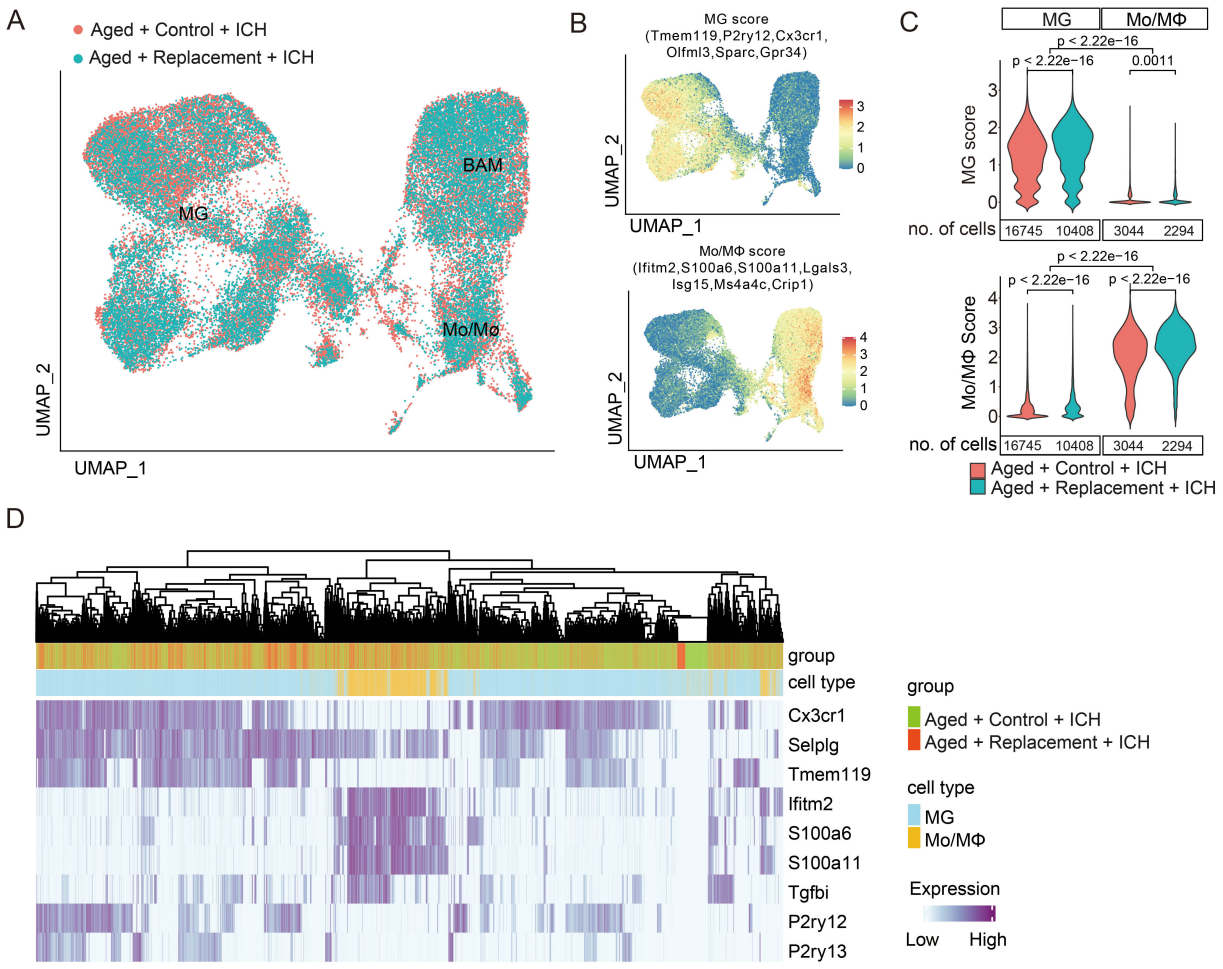
Distinct gene expression profiles of brain injury-associated microglia and monocytes/macrophages. **(A)** UMAP plots demonstrate the distribution of myeloid cells from aged ICH mice treated with or without microglial replacement. **(B)** Distribution of MG and Mo/MΦ “signature” gene scores, which were presented as an average of expression of the selected genes. **(C)** No overlap of a specific “signature” between MG and Mo/MΦ populations was shown by the density plots of MG and Mo/MΦ scores across the two cell populations, *t*-test. **(D)** Cell hierarchical clustering showing cell distribution features according to the expression of reported microglia and macrophage markers, two-sided Fisher’s exact test.

Distribution of cells according to the experimental conditions (microglial replacement or not) revealed similar and evenly distributed functional subgroups of MG, Mo/MΦ, and BAM but with distinct distribution features supported by the unsupervised clustering (**Figure 3A**; **Supplementary Figure S5**). There are a total of 12 subclusters were divided into BAM (cluster 0), MG (cluster 1-3), and Mo/MΦ (cluster 4-11). The cell proportions of most clusters decreased in injured brains after microglial replacement (**Supplementary Figure S5**), suggesting that aging microglial replacement markedly influences these myeloid cells in response to brain injury.

By defining MG and Mo/MΦ scores (35) as an average expression levels of genes restricted to and highly expressed in a given population (**Figure 3B**), we further examined whether the “signature” gene expression of microglia and macrophage is modified in injured brains after microglial replacement. A shift was found toward the higher “microglia signature” score in MG from the injured brains with microglial replacement compared to those without microglial replacement (**Figure 3C**). On the contrary, the “macrophage signature” score in Mo/MΦ was lower after microglial repopulation. Still, the “microglia signature” in MG and “macrophage signature” in Mo/MΦ from both conditions were distinguishable from each other, allowing for clear separation of the two cell populations. Using selected markers, we performed hierarchical clustering of cells based on the expression of reported markers of microglia and macrophage, resulting in a clear separation of microglia and Mo/MΦ (**Figure 3D**). Except for one marker gene, *Selplg* was expressed both in microglia and Mo/MΦ (**Figure 3D**). Selplg encodes p-selectin glycoprotein ligand-1 (PSGL-1), a crucial factor in leukocyte recruitment into inflamed tissue (57, 58), has been shown to recruit the infiltration of leukocytes to the ischemic brain tissues (59, 60). This observation indicated that the expression of signature genes is retained in response to brain injury after aging microglial replacement. Altogether, we show that MG undergoes aging replacement associated with increasing of “microglia signature” gene expression, while Mo/MΦ experiences with slight reduction of expression signatures. Both MG and Mo/MΦ retain expression of “signature” genes within injured brains.

### 3.4 Transcriptional networks induced by brain injury are pronounced in resembled young microglia

**Figure 4:**
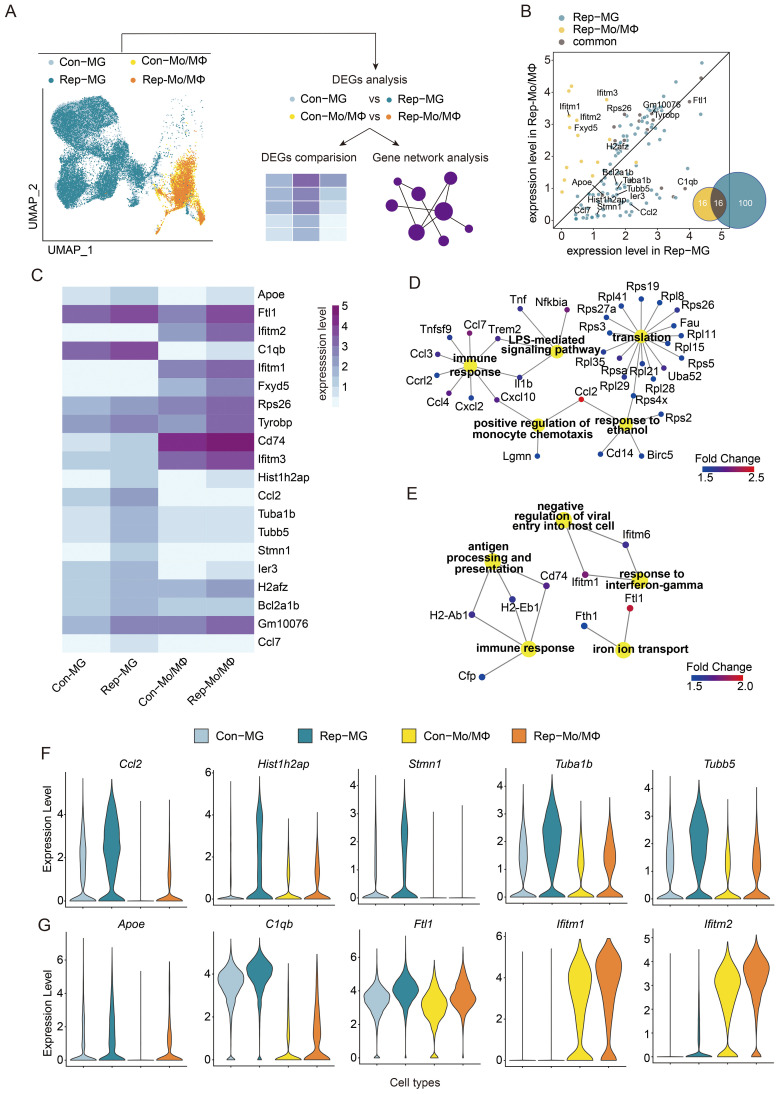
Functional analysis of the comparison of activated microglia or monocytes/macrophages in injured brains between aged ICH mice treated with or without microglial replacement. **(A)** Scheme of the analytical approach. **(B)** Scatter plot depicting expression levels of differentially upregulated genes in microglia (Rep-MG) and Mo/MΦ in replacement group (Rep-Mo/MΦ) when compared to Con-MG and Con-Mo/MΦ, respectively. Venn diagram showing the number of identical and different differentially upregulated genes between Rep-MG and Rep-Mo/MΦ. **(C)** Heatmap showing the comparison of expression of top 10 upregulated genes in Con-MG *vs* Rep-MG and Con-Mo/MΦ *vs* Rep-Mo/MΦ. **(D, E)** Gene Ontology analysis of biological processes for genes upregulated in **(D)** Rep-MG compared to Con-MG and **(E)** Rep-Mo/MΦ compared to Con-Mo/MΦ. **(F, G)** The expression level of selected genes (TOP5; P-value < 0.05, Fold change ≥ 1.5, and min.pct ≥ 0.25) expressed specifically in distinct subpopulations in Rep-MG *vs* Con-MG **(F)** and Rep-Mo/MΦ *vs* Con-Mo/MΦ **(G)**, respectively.

As demonstrated above, the gene expression profiles of MG and Mo/MΦ are distinct. To elucidate their roles in mediating brain injury, we examined the transcriptional networks activated in MG and Mo/MΦ in response to brain injury by analyzing the significantly upregulated genes in Rep-MG and Rep-Mo/MΦ to find genes either common or specific for each subpopulation (**Figure 4A**). The total number of upregulated genes in Rep-MG is much larger than that in Rep-Mo/MΦ (Rep-MG *vs* Rep-Mo/MΦ: 116 *vs* 32; **Figure 4B**), suggesting that the aging microglial replacement mainly influenced the microglia response to injured brains. Then, we found that half of the genes upregulated in the Rep-Mo/MΦ are also expressed by Rep-MG, and their expression is usually higher in Rep-Mo/MΦ than in Rep-MG (**Figures 4B, C**). Among commonly induced genes, we found *Ftl1*, *Rps26*, *Gm10076*, *Tyrobp*, *H2afz*, *Apoe*, and *C1qb*. Rep-MG showed a relative high expression of *Stmn1* and *Ccl7* when compared to Con-MG and Mo/MΦ. In contrast, Rep-Mo/MΦ were characterized by high expression of *Apoe*, *C1qb*, *Ier3*, and *Hist1h2ap* genes when compared to Con-Mo/MΦ (**Figures 4B, C**). However, these relatively highly expressed genes actually exhibit low expression levels at the whole gene expression level (**Figure 4C**), suggesting these genes may partly influence the functions of Rep-MG and Rep-Mo/MΦ.

Next, we performed Gene Ontology (GO) analysis of biological processes on two sets of genes-genes significantly upregulated in Rep-MG (**Figure 4D**) and in Rep-Mo/MΦ (**Figure 4E**) compared to the Controls. The term directly related to the immune function is shared between upregulated genes in Rep-MG and Mo/MΦ but with different cytokines. Gene expression in Rep-MG was enriched in terms “LPS-mediated signaling pathway”, “translation”, “positive regulation of monocyte chemotaxis”, and “response to ethanol”. These results were similar to a previous report that many of the upregulated genes in MG involved in neurodegenerative, ageing or neuroinflammatory processes were upregulated in repopulated MG in old mice (61). In addition, the ethanol response has been shown to be neuroprotection for stroke and traumatic brain injury (62). Whereas terms “antigen processing and presentation” and “iron ion transport” were enriched in Rep-Mo/MΦ. In addition, Rep-Mo/MΦ demonstrated the enrichment of “response to interferon-gamma” genes (Ifitm1 and Ifitm6), which has been shown to promote microglial migration (63) and exacerbate brain injury of stroke (64). In addition, the ifitm1 exhibits antiviral properties (65). Given that stroke-induced immune suppression increases infection risks (66), therefore, interferon pathway upregulation in Rep-Mo/Mϕ may confer benefits for brain recovery. Previous studies have demonstrated that newly repopulated microglia had reduced expression of inflammatory markers (e.g., IL-1β, IL-6, TNF-α and CD86), and upregulation of neuroprotective factors (e.g., CD206, TGF-β, and IL-10) in response to ICH (13) ischemic stroke (67) in ageing. Together, these results indicated that these upregulation pathways in Rep-MG and Rep-Mo/Mϕ might create an attenuated inflammatory milieu in the aged brain in response to brain injury after ICH.

Several shared genes were expressed at a higher level in Rep-MG compared to their levels in Con-MG (**Figure 4F**; **Supplementary Table 3**). Proteins encoded by those genes are involved in immune responses and neuronal functions: CCL2, a member of the monocyte chemokine protein family, induce monocyte infiltration and mediate inflammation to contribute to brain injury after stroke (68); Tuba1b is associated with the immune cell infiltration (69); Tubb5 influence the terminal differentiation and dendritic spine densities of cerebral cortical neurons (70). One gene specifically expressed in Rep-MG is Stmn1, a mitotic gene (47), that has been shown to be involved in the proliferation and differentiation of cancer cells (71), indicating that the repopulated microglia from older mice were proliferative (61). Compared to the genes highly expressed in Rep-MG, few specifically and highly genes were found to be expressed in Rep-Mo/MΦ. One of the relatively highly expressed genes in Rep-Mo/MΦ is Ifitm2 (**Figure 4G**; **Supplementary Table 3**). Such expression patterns may indicate that both MG and Mo/MΦ experience some modification of their immune responses after aging microglial repopulation, with more prominent changes in MG.

### 3.5 The progressive aging trajectory of microglia is reversed after replacement

**Figure 5:**
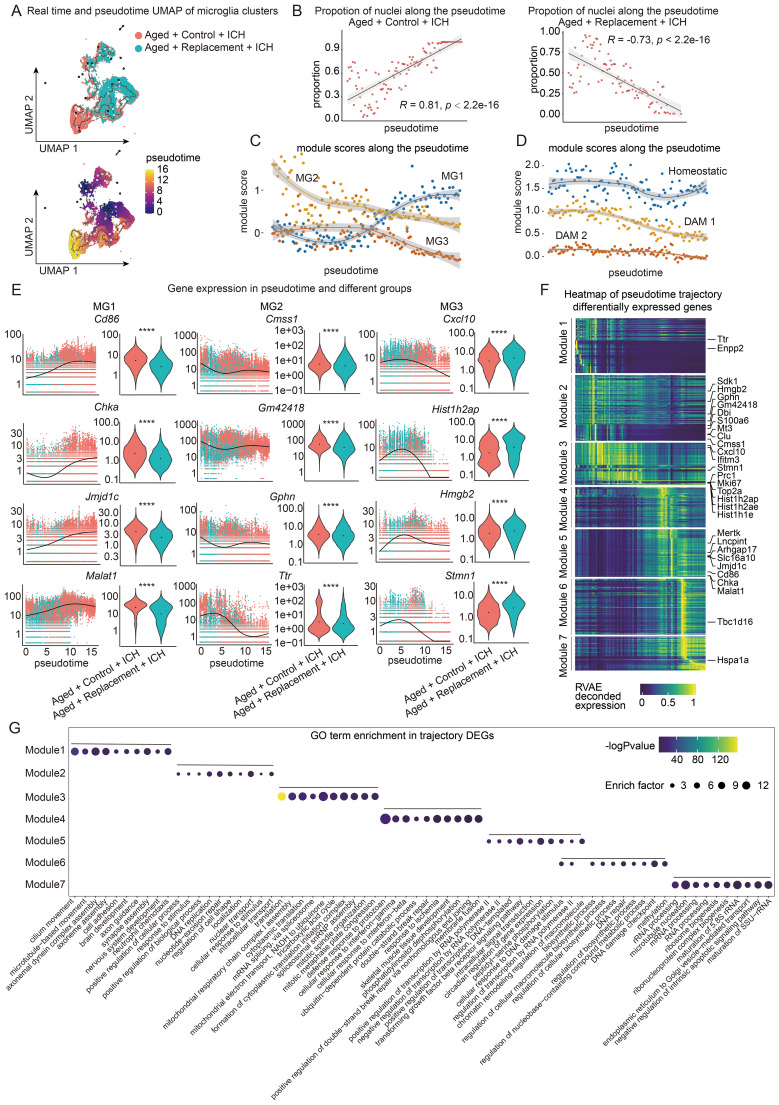
Trajectory analysis of microglia in aged ICH mice. **(A)** Monocle3 pseudotemporal ordering of microglia clusters (n = 27153 nuclei) defining a trajectory signature from Replacement to control nuclei. Nuclei are colored by condition (Aged + Control + ICH and Aged + Replacement + ICH, top) and pseudotime (bottom). **(B)** Scatter-plot showing the proportion of nuclei in Aged + Control + ICH and Aged + Replacement + ICH along the pseudotime timeline, respectively. Pearson correlation of the proportion of aged nuclei and pseudotime timeline, R = 0.81, *P* < 2.2e-16 (two-sided), 95% CI (in gray) in Aged + Control + ICH group and R = -0.73, *P* < 2.2e-16 (two-sided), 95% CI (in gray) in Aged + Replacement + ICH group shown. **(C, D)** Plot showing the module expression score of two kinds of three microglia states (one is MG1, MG2, and MG3 shown in **C** and another is homeostatic, DAM 1, and DAM 2 shown in **D**). The darker lines are the local regression result for time bins sized 0.15, with the gray shadow depicting the 95% CIs. **(E)** Kinetics plot showing the relative expression of representative genes for MG1, 2, and 3 states (left). The lines approximate expression along the trajectory using polynomial regressions. Violin plots of gene expression and the results of MAST with a random effect for a sample of origin and sequencing depth, with *t*-test *P* values (****P < 0.0001) (right). **(F)** Heat map showing modules of trajectory DEGs (t-DEGs) in the microglia cluster (n= 17064 genes). The expression value is an RVAE decoded expression. The genes were grouped into 7 modules after ranking by RVAE-decoded expression. Module 1 (2745 genes), module 2 (3294 genes), module 3 (2119 genes), module 4 (2021 genes), module 5 (2384 genes), module 6 (2796 genes), and module 7 (1705 genes). **(G)** Dot plot showing the terms of the top ten GO biological processes for genes in each module.

Then, we performed pseudotemporal ordering of nuclei from the microglia clusters by using Monocle3 (29) to uncover changes in these cells over time (**Figure 5A**). The trajectory accurately captures the transition from Con-MG (aged) to Rep-MG (young) nuclei, suggesting a reversing progression toward microglia after the replacement and a significant increase or decrease in the proportion of Con-MG (aged) or Rep-MG (young) nuclei across pseudotime, respectively (**Figures 5A, B**).

Using the same markers of their clusters, the key gene sets were aggregated and a module score along the pseudotime trajectory was plotted to investigate how the identified three microglia clusters. Overall, there was a significant increase in the module score for the MG1 module over pseudotime, which was consistent with the changes in the proportion of MG1 between the Replacement and Control groups (**Figure 5C**). In contrast, we observed a nearly linear decrease in the MG2 module score near the beginning of pseudotime and a decrease in the MG3 module score near the medial of pesudotime (**Figure 5C**). Such expression-changing features suggest an important role of involving in the contribution of brain injury of different microglial subgroups over time.

In addition, since changes in disease-associated microglia (DAM) have also been implicated in both brain aging and neurodegeneration (72), we examined whether and how DAM genes change as a function of pseudotime under brain injury. Similarly, the expression of key genes from three microglia gene sets identified in the literature (72) were aggregated, homeostatic microglia (homeostatic), TREM2 independent stage 1 DAM (DAM 1), and TREM2 dependent stage 2 DAM (DAM 2), and the pseudotime trajectory was plotted (**Figure 5D**). A slight decrease in the module score for the homeostatic module was found in the second half of pseudotime, suggesting a loss of maintenance of healthy microglia in the Control groups. In contrast, we observed a significant decrease in the DAM 1 disease module score over time. The DAM 2 module does not seem to change and the module score remains low throughout pseudotime, suggesting microglial repopulation may not influence this subgroup of microglia to participate in the regulation of brain injury.

To further understand the role of these gene modules in Con-MG and Rep-MG, gene expression across pseudotime between the two conditions was visualized (**Figure 5E**). While Rep-MG (young) generally cluster earlier in pseudotime (pseudotime 0.0 through 7.5), these genes expressed in Con-MG (aged) are distributed throughout pseudotime and mainly in the later pesudotime (**Figure 5E**; **Supplementary Figure S6**, **S7**). Therefore, microglia in the control group remain in the aging state in response to brain injury, while microglia in the replacement group show an increased heterogeneity representing a progressive young state in response to brain injury.

To fully capture the trajectory of changes in gene expression, Moran’s I test on MG genes was performed and 17064 statistically significant trajectory-dependent genes were found (**Supplementary Table S1**). To characterize their expression dynamics along pseudotime by applying RVAgene (73), an autoencoder neural network framework for reconstructing and smoothing pseudotime-dependent gene expression. Then, a heat map was made to visualize the recurrent variational autoencoder (RVAE) decoded expression along pseudotime, and the genes were manually grouped into 7 modules according to their pseudotemporal expression patterns (**Figure 5F**). For example, genes in module 1 are highly expressed in early pseudotime while genes in module 7 are expressed in late pseudotime. Finally, GO analysis was performed to understand the biological processes of these genes enriched in each module (**Figure 5G**). Notably, gene modules are transitioning through pseudotime from positive regulation of neuronal functions (e.g., axonal guidance, synapse assembly) to biological process, gene regulation, immune responses, and finally to negative regulation of intrinsic apoptotic signaling pathway.

### 3.6 The progressive aging trajectory of monocytes/macrophages is slightly reversed after microglial replacement

**Figure 6:**
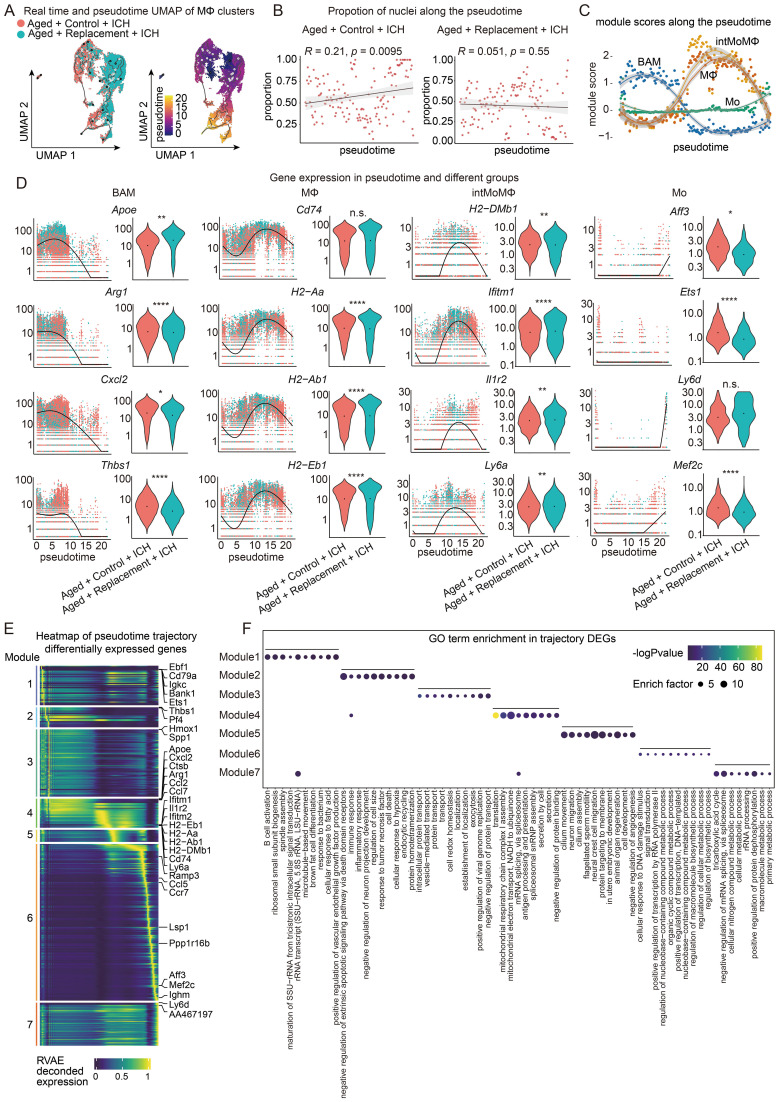
Trajectory analysis of macrophages in aged ICH mice. **(A)** Monocle3 pseudotemporal ordering of macrophage clusters (n = 17917 nuclei) defining a trajectory signature from Replacement to control nuclei. Nuclei are colored by condition (Aged + Control + ICH and Aged + Replacement + ICH, top) and pseudotime (bottom). **(B)** Scatter-plot showing the proportion of nuclei in Aged + Control + ICH and Aged + Replacement + ICH along the pseudotime timeline, respectively. Pearson correlation of the proportion of aged nuclei and pseudotime timeline, R = 0.21, *P* = 0.0095 (two-sided), 95% CI (in gray) in Aged + Control + ICH group and R = 0.051, P = 0.55 (two-sided), 95% CI (in gray) in Aged + Replacement + ICH group shown. **(C)** Plot showing the module expression score of four macrophage states (Mo, MΦ, intMoMΦ, and BAM). The darker lines are the local regression result for time bins sized 0.15, with the gray shadow depicting the 95% CIs. **(D)** Kinetics plot showing the relative expression of representative genes for Mo, MΦ, intMoMΦ, and BAM states (left). The lines approximate expression along the trajectory using polynomial regressions. Violin plots of gene expression and the results of MAST with a random effect for a sample of origin and sequencing depth, with *t*-test *P* values (****P < 0.0001, **P < 0.01, *P < 0.05, and n.s. indicates non-significant) (right). **(E)** Heat map showing modules of trajectory DEGs (t-DEGs) in the macrophage cluster (n= 13368 genes). The expression value is an RVAE decoded expression. The genes were grouped into 7 modules after ranking by RVAE decoded expression. Module 1 (1437 genes), module 2 (728 genes), module 3 (2447 genes), module 4 (1056 genes), module 5 (443 genes), module 6 (5694 genes), and module 7 (1563 genes). **(F)** Dot plot showing the terms of the top ten GO biological processes for genes in each module.

Using the same analytic strategy as aforementioned, we further uncovered the changes in monocytes/macrophages over time (**Figure 6A**). The trajectory accurately captures the transition from young to aged nuclei in the Replacement group, while there is no obvious transition in the Control groups, suggesting a reversing progression toward monocytes/macrophages after the replacement. Only a slight increase in the proportion of nuclei across pseudotime was found in the Control group and no significant changes in the Replacement group (**Figure 6B**).

We further investigated how the clusters of Mo, intMoMΦ, MΦ, and BAM change as a function of pesudotime. Their marker gene sets were aggregated to plot a module score along the pseudotime trajectory. The results showed that there was a significant rapid increase in the module score for both the intMoMΦ and MΦ modules near the medial of pseudotime but then subsequently restored sharply back to earlier levels at the end of pseudotime (**Figure 6C**). In contrast, we observed a rapid decrease in the BAM module score near the medial of pseudotime (**Figure 6C**). The Mo module score remains relatively stable throughout pseudotime with a slight increase at the end of pseduotime. These results together suggest that the expression features of BAM, intMoMΦ, and MΦ may be pronounced changed in response to brain injury after microglial replacement.

To further understand the role of these gene modules in the four clusters, gene expression across pseudotime between the control and replacement conditions was visualized (**Figure 6D**). Both Con-BAM and Rep-BAM generally cluster earlier in pseudotime (pseudotime 0.0 through 10), while the genes expressed in intMoMΦ and MΦ both in control and replacement groups are distributed throughout pseudotime (**Figure 6D**; **Supplementary Figure S8**). Overall, the genes expressed in Mo are sparsely distributed over time. Therefore, BAM, intMoMΦ, and MΦ in the injured brains are easily influenced by microglial replacement, especially for the BAM.

To fully capture the trajectory of changes in gene expression, Moran’s I test on these genes was performed and 13368 statistically significant trajectory-dependent genes were found (**Supplementary Table S2**). Similar to the MG, the genes expressed in monocytes/macrophages along pseudotime were also manually grouped into 7 modules according to their pseudotemporal expression patterns (**Figure 6E**). For example, genes in module 1 are highly expressed in early and late pseudotime, genes in modules 2 and 3 are highly expressed in the first half of pesudotime, and genes in module 7 are expressed almost in late pseudotime. Finally, GO analysis was performed to understand the biological processes of these genes enriched in each module (**Figure 6F**). Notably, gene modules are transitioning through pseudotime from immune response to biological process, gene regulation, neuronal functions, and finally to metabolic process.

## 4 Discussion

The molecular changing features of resembling young microglia in aged mice in response to brain injury and whether this change influences the other myeloid cells remain unclear. In the present study, we performed snRNA-seq to dissect the cellular and molecular signatures of microglia and other myeloid cells in injured brains of aged ICH mice after microglial replacement. A major finding of this study is that there are considerable changes in the proportion of subclusters of myeloid cells and obvious molecular changes in MG heterogeneity while slight Mo/MΦ changes, indicating that the changes in myeloid cell composition and gene expression characteristics may be beneficial to alleviate brain injury in general when aged microglia are replaced. Importantly, trajectory analysis showed that most of the myeloid cells exhibited a signature of a young state with more pronounced changes in microglia, which further suggested a potential neuroprotective mechanism of aged microglial repopulation against brain injury. These findings indicate the potential neuroprotective mechanism of resembling young-like microglia in aged brain injury and provide resourceful data for further exploration of key targets for the treatment of aged brain injury.

The factors influencing the infiltration of peripheral immune cells into injured brains are quite complicated. In this study, we found that even under the same brain injury in aged mice, the cell proportions of myeloid cells (MG, Mo/MΦ, and BAM) underwent remarkable changes induced by aged microglial replacement, indicative of their differing contributions to brain injury in aged mice. Among these identified cell types and subclusters, the cell proportion of BAM, MG1, and MG3 changed most dramatically after replacement. For example, the proportion of the BAM cluster, which has been shown to contribute to ischemic stroke and cerebral amyloid angiopathy and cognitive impairment (74, 75), increased from 16.78% in control groups to 20.7% in replacement groups. These results indicate that such changed cell proportions of myeloid cells in injured brains of aged mice after microglial replacement may be the result of the complicated microenvironment of aged brain injury that influenced and determined, rather than solely being influenced by the resemblance to young-like microglia , since microglial replacement has obvious indirect or off-target effects evidenced by influencing other myeloid cells (76, 77). However, the molecular mechanisms underlying microglial replacement-mediated influences on myeloid cell phenotypes remain largely unexplored but constitute an important research topic, meriting future in-depth exploration.

Although significant expression pattern changes were observed in myeloid cells (particularly BAMs) after microglial replacement compared to controls, which might influence the detrimental effects of BAMs on brain injury because previous studies showed that BAMs undergo dynamic transcriptional shifts, participate in inflammatory pathway regulation (78) and promote stroke-related neurological impairment (79). The pathophysiological roles of BAMs remain incompletely elucidated. Given their unique localization at CNS borders, they likely mediate physiological functions through cooperative interactions with other brain cells (80). Under pathological conditions, BAMs may undergo genetic transformations, proliferation, and migration, contributing to brain disease processes including ischemic stroke (80). However, determining whether post-replacement gene expression shifts in BAMs reflect functional rejuvenation or transient transcriptomic changes proves challenging-despite observed pseudotemporal expression differences-necessitating further investigation.

Another important and interesting finding refers to the trajectory analysis of microglia and monocytes/macrophages in brain injury after microglial replacement. As expected, both MG and Mo/MΦ in the injured brain after microglial replacement experienced a transition from an aged to a young state across pseudotime with more pronounced for MG and slight changes for Mo/MΦ, while little transition was found in control groups. This trajectory analysis provides molecular evidence of aged microglial elimination withdrawal giving birth to young-like microglia. In addition, the results of gene expression across pseudotime showed that the expression of genes, such as Cd86 and Chka, in Rep-MG1 generally clusters earlier in pseudotime, while in Con-MG1, they are distributed mainly in the later pesudotime and the overall expression level of these genes was decreased in replacement groups when compared to control groups, further indicating a shift from aging state of microglia in control group toward young state in replacement group. Cd86 is a classic marker of the M1 phenotype, which has been widely considered to contribute to brain injury (81) and is significantly increased in brains after stroke (82). Choline kinase alpha (Chka) acts as a protein kinase to promote lipolysis of lipid droplets (83), a process linked to microglial dysfunction and pro-inflammatory states in the aging brain (84) and Alzheimer’s disease (85). These results suggested that the reduction of the expression of these genes in MG1 is beneficial to brain injury after microglial replacement.

However, the proportion of the cluster MG3 increased from 5.78% in the control groups to 9.6% in the replacement groups and similar expression signatures of genes, like Cxcl10 and Hmgb2, across pseudotime were also found in MG3. These genes seem to be detrimental to brain injury. For example, the upregulated expression of the inflammatory chemokine Cxcl10, which is an interferon (IFN) type I stimulated gene, has been reported in models of ischemic brain injury, with Cxcl10-expressing microglia localized in the infarct border at the acute phase and in the lesioned tissue at later phase (47). In addition, the elevation in blood CXCL10 was independently associated with worse outcomes of patients with ICH (86), potentially via impairing the synaptic plasticity (87). The high mobility group protein B2 (Hmgb2) was identified as a microglial pro-inflammatory mediator (88). Therefore, the significantly increased proportion of MG3 in the replacement group and the detrimental roles of some cluster-specific genes are contradictory to the neuroprotective effect of microglial replacement. Nevertheless, the roles of some genes in MG3 , such as Hist1h2ap and Stmn1, in regulating microglial biofunction and brain injury remain largely unknown and deserve further investigation.

This study has several limitations. First, the exclusive use of male aged mice to investigate molecular changes in repopulated microglia post-brain injury may limit the generalizability of findings , given documented sex differences in behavioral, cellular, and molecular characteristics. Future studies should explicitly address sex-dependent variations in microglial replacement. Second, although we focused on aged mice’s differential response mechanisms, the lack of young mouse controls hindered comparative analysis of repopulated microglia’s molecular mechanisms across lifespan stages. Third, while injury-induced transcriptional changes were detected in repopulated microglia (Rep-MG), the functional consequences and underlying mechanisms demand further investigation in aging contexts.

## 5 Conclusion

In conclusion, by analyzing the cellular and molecular signatures of myeloid cells in response to brain injury at single-cell resolution after microglial replacement, this study identifies cell type-specific immune signatures and cell fate changes following microglial replacement , and provides a resource data for identifying potential therapeutic targets to reduce brain injury caused by aged microglia after stroke.

## Data Availability

The datasets presented in this study can be found in online repositories. The names of the repository/repositories and accession number(s) can be found in the article/**Supplementary Material**.
