# Supplementary material for: Single-nucleus RNA sequencing reveals the specific molecular signatures of myeloid cells responding to brain injury after microglial replacement
Source: Front Immunol. 2025 Jul 24;16:1625673. doi: 10.3389/fimmu.2025.1625673 (PMC12328185; doi:10.3389/fimmu.2025.1625673)
Supplement: Supplementary file 1 [file Table1.docx]

**
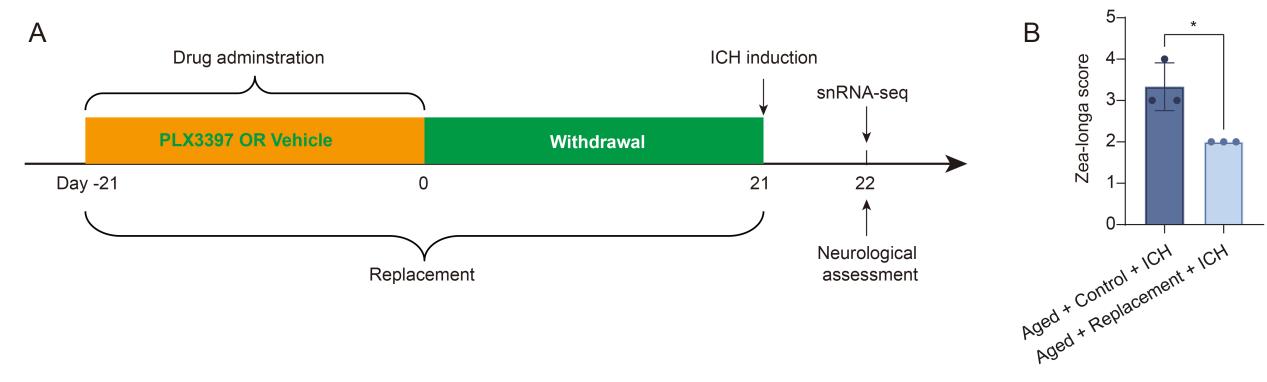
**

**Supplementary Figure S1. Microglial replacement in the aged brain.** (**A)** A Flow chart illustrates drug administration and experimental design. Groups of aged mice received 21 days of PLX3397 treatment by gavage and then followed by three weeks of withdrawal before ICH induction. ICH induction, neurological assessment, and tissue collection were conducted at indicated time points. (**B)** The results of Zea-longa score of aged mice treated with or without PLX3397 at 1 day post-ICH, with *t*-test *P* values, **P* < 0.05 (n = 3).

**
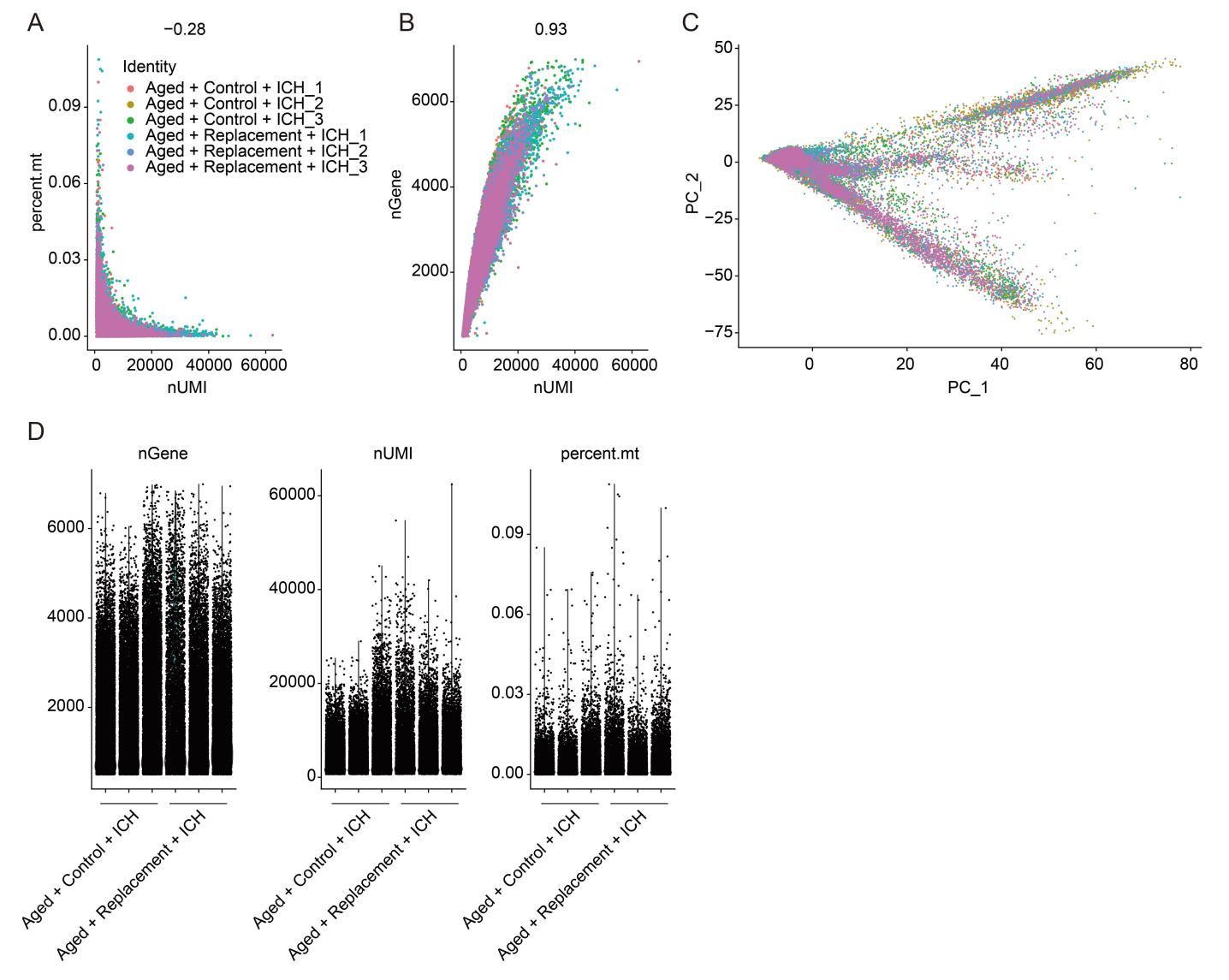
Supplementary Figure S2. Single-nucleus RNA-sequencing of perihematomal brain tissues.** (**A, B)** The scatter plot shows the number of UMIs detected in cells (x-axis) in relation to mitochondrial gene proportion (y-axis) (**A**) and the number of genes (y-axis) (**B**). The colors representing samples, and the number above the plot are the value of the correlation coefficient between the x- and y-axes. (**C)** The result of PCA-based reduced dimension clustering. The colors represent samples. (**D)** The boxplot shows the number of genes, UMI counts, and mitochondrial proportions for each cell in each sample.

**
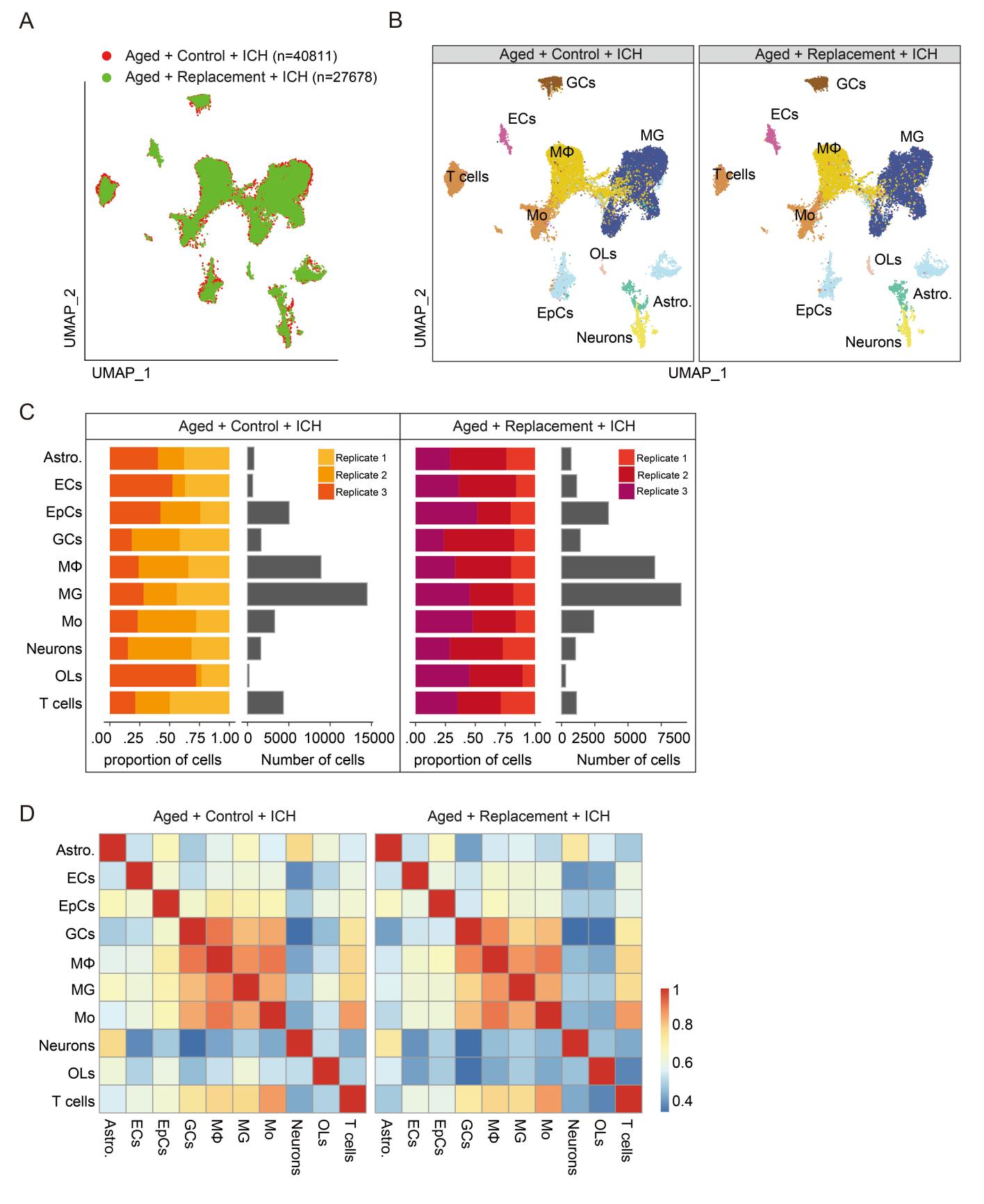
Supplementary Figure S3. Comparison of the snRNA-seq replicates.** (**A)** UMAP shows a uniform distribution of cells from injured brains of aged ICH mice with or without treatment with PLX3397. (**B)** UMAPs demonstrate that all obtained clusters were present for both control and replacement conditions. (**C)** The proportion of cells from injured brains of aged ICH mice with or without treatment with PLX3397 was comparable across all obtained clusters. (**D)** Correlation heatmaps comparing gene expression profiles of the identified clusters between Aged + Control + ICH (left) and Aged + Replacement + ICH groups (right).

**
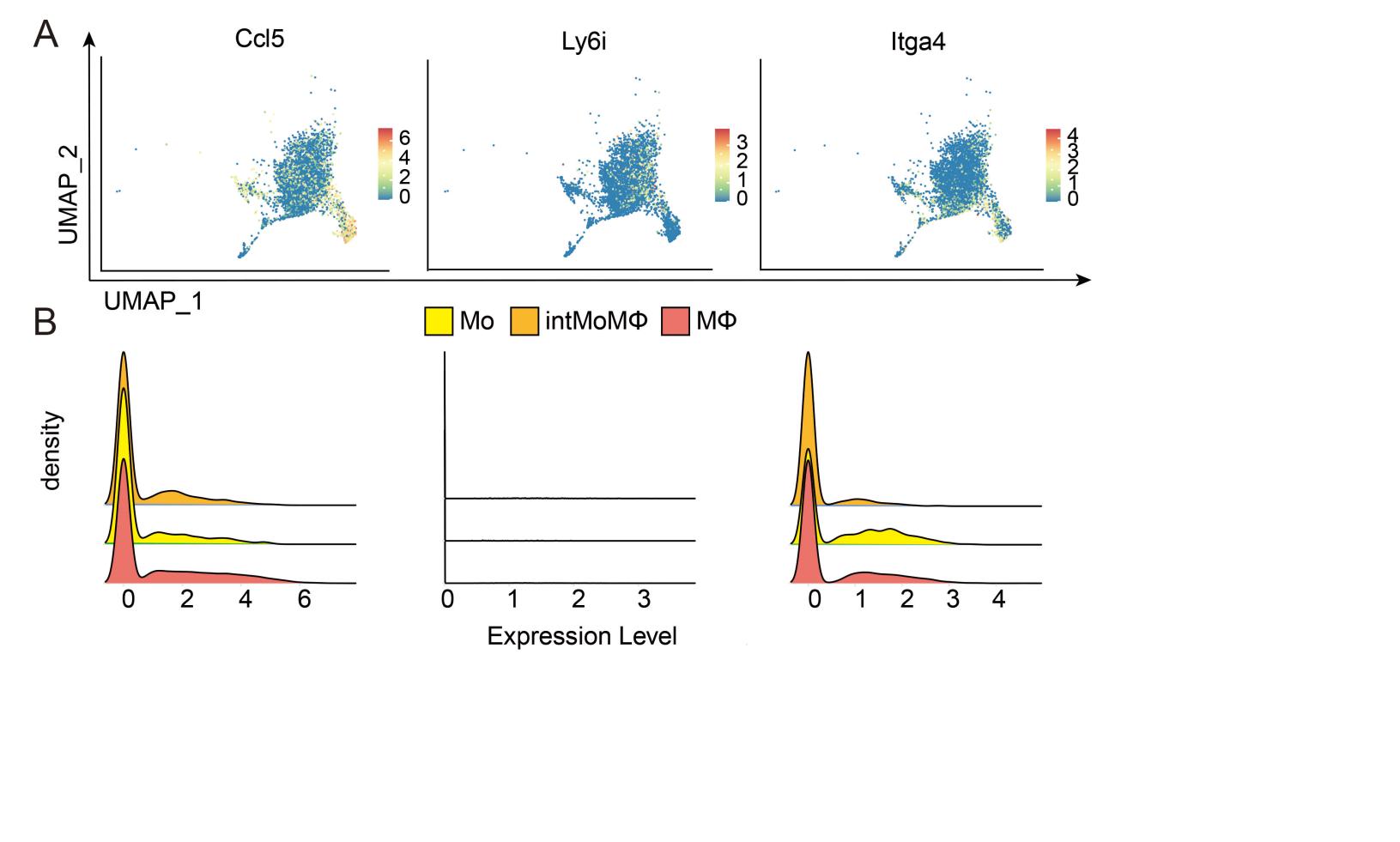
Supplementary Figure S4. The distribution and expression level of the marker genes.** Feature (**A**) and density plots (**B**) depicting distribution and expression level of the marker genes, Ccl5, Ly6i, and Itga4 to discriminate the subpopulations of monocytes (Mo), monocyte-macrophage intermediate (intMoMΦ), and macrophage (MΦ).

**
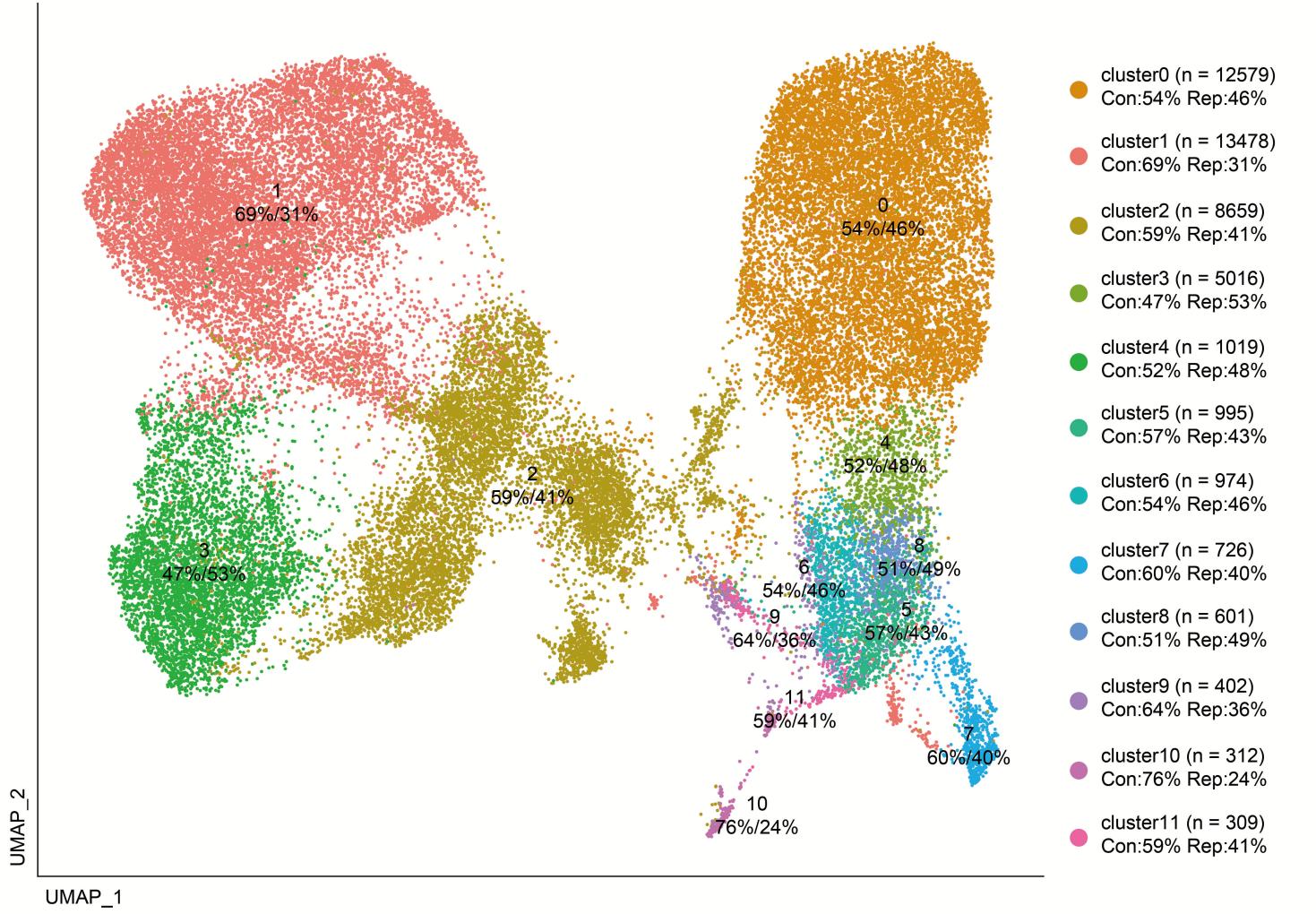
Supplementary Figure S5. Clustering of cells identified as Microglia, Monocytes/Macrophages, and BAMs from all conditions.** UMAP plot showing the results of unsupervised clustering of cells from three subpopulations of interest (corresponding to Figure 2A) from all 6 samples. Clusters are marked with different colors and the number of cells assigned to each cluster (n) is depicted in the legend. The shown percentages correspond to fractions of cells originating from control and replacement-treated samples, respectively.

**
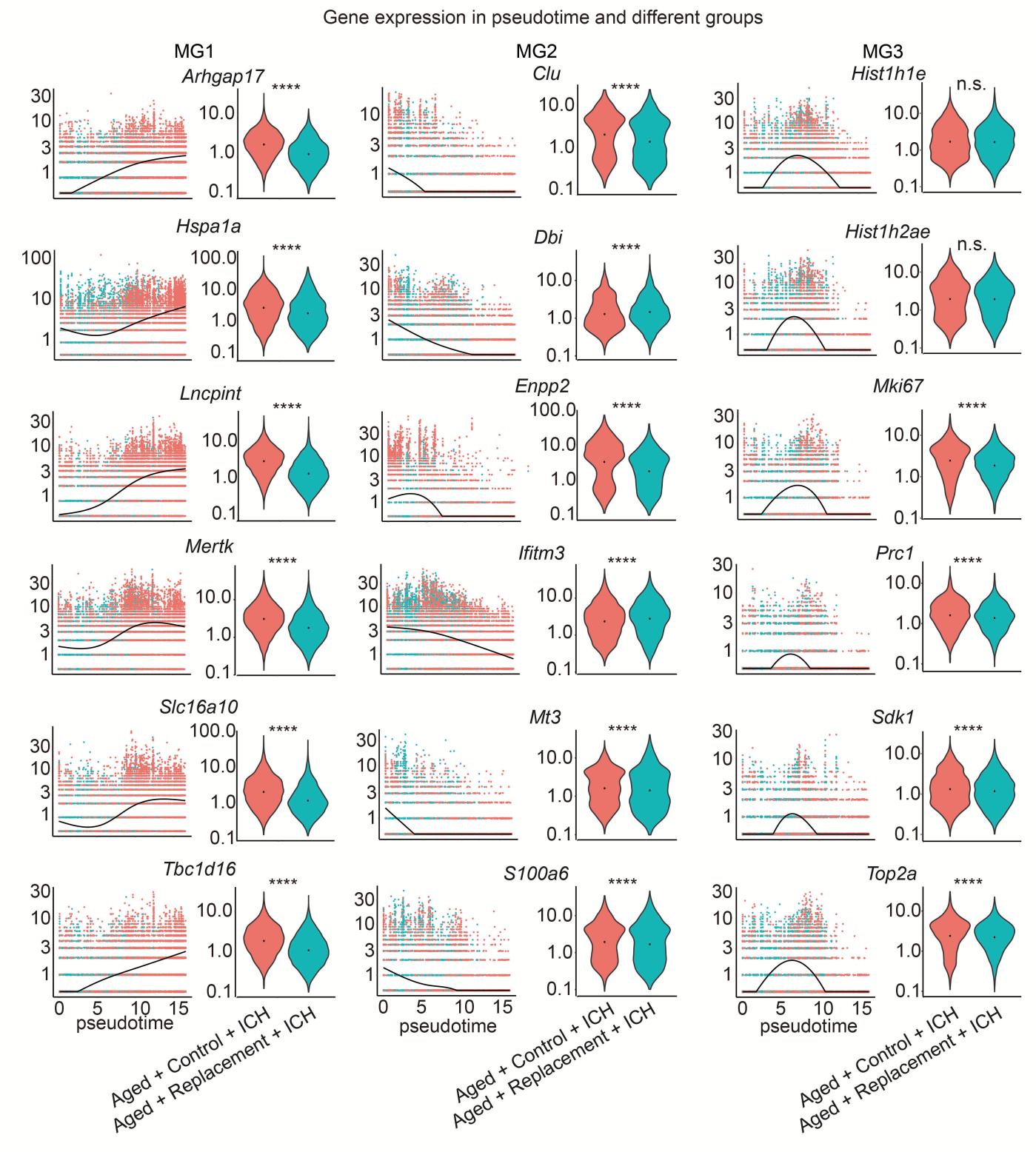
Supplementary Figure S6.** Kinetics plot showing the relative expression of representative genes for MG1, 2, and 3 states (left). The lines approximate expression along the trajectory using polynomial regressions. Violin plots of gene expression and the results of MAST with a random effect for a sample of origin and sequencing depth, with *t*-test *P* values (right). *****P* < 0.0001 and n.s. indicates non-significant.

**
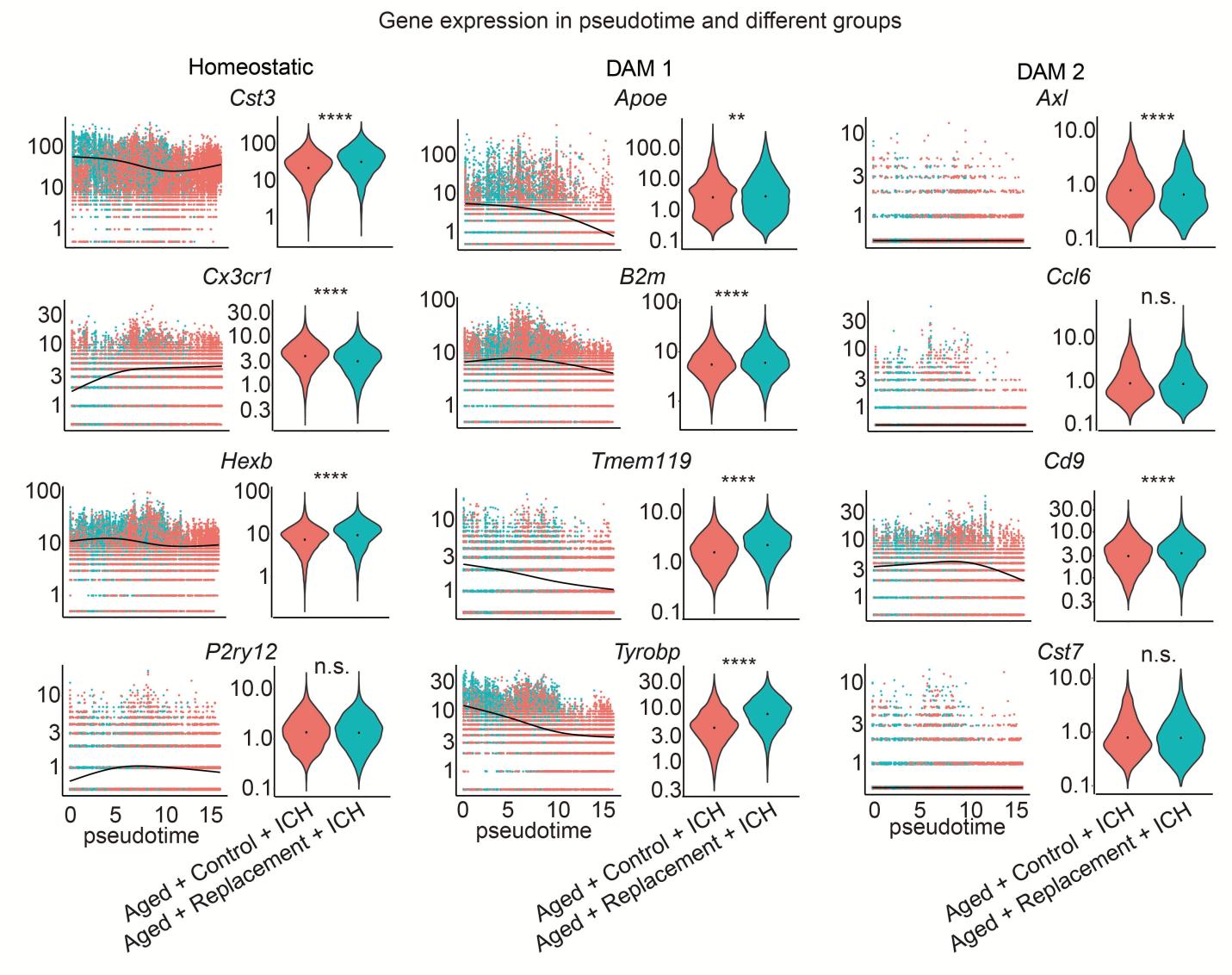
Supplementary Figure S7.** Kinetics plot showing the relative expression of representative genes for homeostatic, DAM 1, and DAM 2 states (left). The lines approximate expression along the trajectory using polynomial regressions. Violin plots of gene expression and the results of MAST with a random effect for a sample of origin and sequencing depth, with *t*-test *P* values (right). *****P* < 0.0001, ***P* < 0.01, and n.s. indicates non-significant.

**
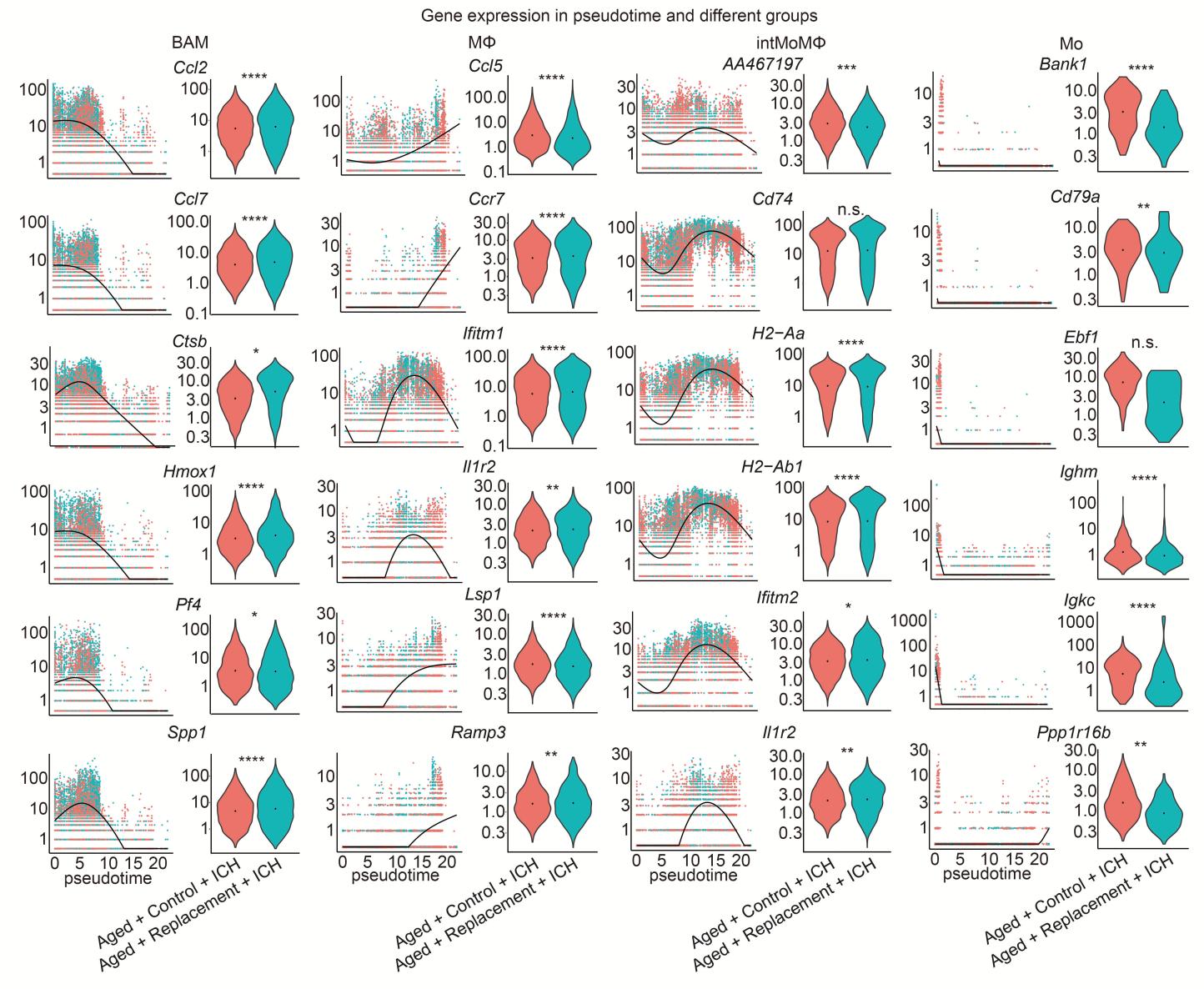
Supplementary Figure S8.** Kinetics plot showing the relative expression of representative genes for Mo, MΦ, intMoMΦ, and BAM (left). The lines approximate expression along the trajectory using polynomial regressions. Violin plots of gene expression and the results of MAST with a random effect for a sample of origin and sequencing depth, with *t*-test *P* values (right). *****P* < 0.0001, ****P* < 0.001, ***P* < 0.01, **P* < 0.05, and n.s. indicates non-significant.
